# Single‐Phase *L*1_0_‐Ordered High Entropy Thin Films with High Magnetic Anisotropy

**DOI:** 10.1002/advs.202308574

**Published:** 2024-06-28

**Authors:** Willie B. Beeson, Dinesh Bista, Huairuo Zhang, Sergiy Krylyuk, Albert V. Davydov, Gen Yin, Kai Liu

**Affiliations:** ^1^ Physics Department Georgetown University Washington DC 20057 USA; ^2^ Materials Science and Engineering Division National Institute of Standards and Technology Gaithersburg MD 20899 USA; ^3^ Theiss Research, Inc. La Jolla CA 92037 USA

**Keywords:** high entropy alloy, magnetic anisotropy, rapid thermal annealing

## Abstract

The vast high entropy alloy (HEA) composition space is promising for discovery of new material phases with unique properties. This study explores the potential to achieve rare‐earth‐free high magnetic anisotropy materials in single‐phase HEA thin films. Thin films of FeCoNiMnCu sputtered on thermally oxidized Si/SiO_2_ substrates at room temperature are magnetically soft, with a coercivity on the order of 10 Oe. After post‐deposition rapid thermal annealing (RTA), the films exhibit a single face‐centered‐cubic phase, with an almost 40‐fold increase in coercivity. Inclusion of 50 at.% Pt in the film leads to ordering of a single *L*1_0_ high entropy intermetallic phase after RTA, along with high magnetic anisotropy and 3 orders of magnitude coercivity increase. These results demonstrate a promising HEA approach to achieve high magnetic anisotropy materials using RTA.

## Graphical abstract

High entropy alloy (HEA) thin films are explored to achieve high magnetic anisotropy phases. Sputtered FeCoNiMnCu thin films exhibit a single phase and a 40‐fold coercivity increase after rapid thermal annealing (RTA). Inclusion of Pt leads to a single *L*1_0_ phase after RTA with 3 orders of magnitude coercivity increase, demonstrating a promising approach toward novel magnetic HEAs.

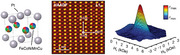

## 1 Introduction

High entropy alloys (HEAs) are a class of materials traditionally defined to contain five or more elements that may exist as stable or metastable single phases due to their high configurational entropy.^[^ ^1^ ^]^ The entropy‐stabilization effect implies a vast number of unexplored material systems with potential to exhibit unconventional properties. So far HEA studies have predominantly focused on exceptional mechanical properties, namely combinations of strength, hardness, and ductility.^[^ ^1^, ^2^ ^]^ In recent years, however, a number of systems have been reported that exhibit other attractive properties and functionalities, which are suitable for hydrogen storage,^[^ ^3^ ^]^ thermoelectrics,^[^ ^4^ ^]^ superconductivity,^[^ ^5^ ^]^ magnetocalorics,^[^ ^6^ ^]^ and soft magnet applications.^[^ ^7^ ^]^ Magnetic studies make up comparatively little of the published literature, and most HEA studies have been limited to bulk systems.

A prospect which remains largely unexplored is the design of HEAs with high magnetic anisotropy, which have critical applications in magnetic recording and permanent magnets.^[^ ^8^ ^]^ As the search for new rare‐earth‐free high anisotropy materials has mostly exhausted the pool of binary and ternary alloy compositions, an emerging opportunity is the arena of high entropy materials, which may hold unique electronic structures favorable to high magnetic anisotropy. Most known HEAs fall in the category of soft magnetic materials, with coercivities below 100 Oe.^[^ ^9^ ^]^ However, coercivities as high as 1200 Oe have been reported in certain HEA systems.^[^ ^10^ ^]^ The coercivity is highly influenced by microstructure which may differ significantly amongst HEA systems depending on the fabrication conditions. The bulk of HEA studies focus predominantly on the maximum entropy solid solutions at the compositional center, which generally consist of uniform chemical disorder within cubic crystal structures, while low‐symmetry crystal structure and chemical order play an important role in magnetocrystalline anisotropy. However, it is not necessary that HEAs be fully disordered, as configurational entropy can be a dominant term in the free energy as long as the number of elements is large relative to the number of unique lattice sites. Certain HEA compositions may exhibit long‐range chemical order through the emergence of multiple sublattices, analogous to high entropy ceramics,^[^ ^11^ ^]^ leading to so‐called “high entropy intermetallic” (HEI) compounds.^[^ ^12^ ^]^ Previously, transition metal alloys with high anisotropy, such as the *L*1_0_ phases of FePt and FePd have been achieved by annealing of equiatomic solid solution pre‐cursors.^[^ ^13^ ^]^ In the case of metastable high anisotropy phases such as *L*1_0_ FeNi, unconventional annealing techniques have been employed.^[^ ^14^ ^]^ In particular, rapid thermal annealing (RTA) of transition metal alloy films on Si/SiO_2_ is an effective means of inducing high anisotropy phase formation in solid solution precursors, in part due to the substrate‐induced strain.^[^ ^15^ ^]^

In this study, we experimentally demonstrate two thin film HEAs with large coercivity and magnetic anisotropy achieved by RTA. We show that RTA treatment of equiatomic FeCoNiMnCu thin films leads to ≈40‐fold increase in coercivity. By introduction of 50 at.% Pt, we realize an *L*1_0_ HEI phase of (FeCoNiMnCu)Pt with perpendicular magnetic anisotropy resulting from a reduced lattice symmetry and strong spin‐orbit coupling. A significant increase of coercivity, by 3 orders of magnitude, is observed.

## 2 Results and Discussion

**Figure 1:**
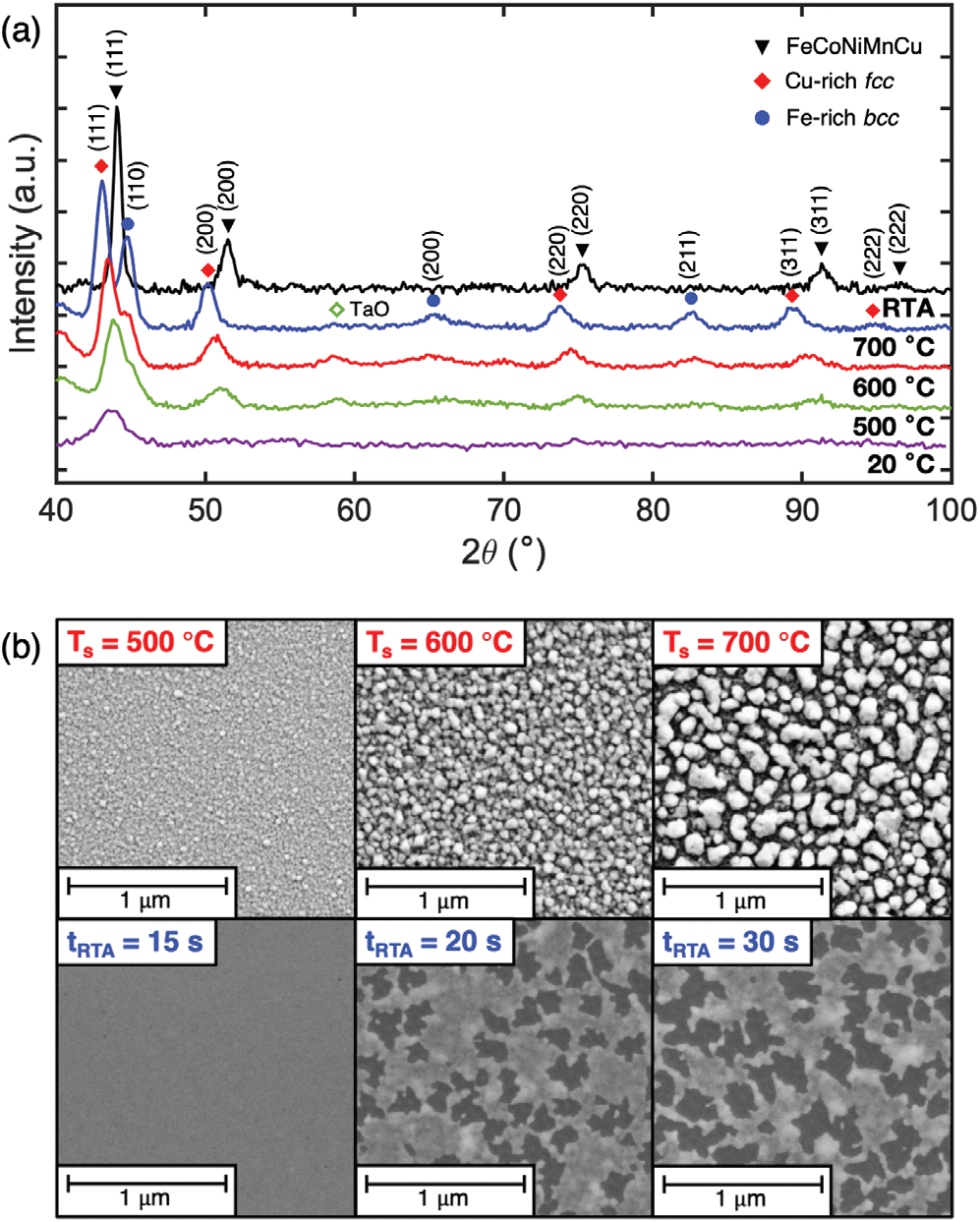
XRD and SEM study of FeCoNiMnCu films grown under different conditions. a) GIXRD scan for 50 nm FeCoNiMnCu films sputtered at various substrate temperatures of 20, 500, 600, and 700 °C (bottom to top), and sputtered at 20 ° C and annealed by RTA at 600 °C for 30 s (top curve). b) SEM images for (top) 50 nm films sputtered at substrate temperatures of 500, 600, 700 °C and (bottom) 13 nm films after RTA at 600 °C for 15, 20, and 30 s.

FeCoNiMnCu films were sputtered from a composite target (Experimental Methods), and a composition of Fe_0.19_Co_0.18_Ni_0.20_Mn_0.18_Cu_0.25_ was confirmed by energy dispersive X‐ray (EDX) microanalysis. Grazing incidence XRD (GIXRD) patterns of the 50 nm FeCoNiMnCu films sputtered at various substrate temperature (T_s_) are shown in **Figure** **1**a, along with those sputtered at 20 °C and treated with RTA for 30 s at 600 °C. The films sputtered at 20 °C exhibit only one peak at ≈44° with relatively low intensity. A similar pattern is seen in the films grown at 350 °C (Figure S2, Supporting Information). For the film grown at T_s_ = 500 °C, new peaks are observed, corresponding to a face‐centered‐cubic (*fcc*) phase with lattice parameter of 3.58 Å and a body‐centered‐cubic (*bcc*) phase with lattice parameter of 2.84 Å. With further increase of T_s_, the peak intensities increase overall and the *fcc* (111) and *bcc* (110) peaks become more separated in 2θ, revealing the increasing chemical segregation between the two phases. At T_s_ = 700 °C, the lattice constants for the *fcc* and *bcc* phases reach 3.63 and 2.86 Å, respectively, close to the bulk values of *fcc* Cu (3.615 Å) and *bcc* Fe (2.867 Å). Considering that Fe‐Cu has a binary mixing enthalpy ΔH_mix_ of 12.9 kJ mol^−1^, which is the most positive of all binary pairs in the FeCoNiMnCu system (Table S1, Supporting Information), we expect it to be a dominant driving force of phase separation.^[^ ^16^ ^]^ Based on this, we consider the separated phases most likely to be a Cu‐rich *fcc* phase and an Fe‐rich *bcc* phase. In the films grown at 500, 600, and 700 °C, there are additional peaks just below 40° and below 60° which originate from Ta capping layer used for their growth.

In contrast, the films sputtered at 20 °C and 350 °C with post‐deposition RTA at 600 °C for 30 s exhibit a single‐phase *fcc* pattern (Figures S1 and S2, Supporting Information). This is in agreement with previous reports of bulk FeCoNiMnCu high entropy alloys^[^ ^6^, ^17^ ^]^ as well as the phase formation criteria based on the valence electron concentration (VEC).^[^ ^18^ ^]^ The formation of single‐phase *fcc* in the RTA film, as opposed to multi‐phase *fcc* + *bcc*, demonstrates the advantage of the RTA method in suppressing growth of secondary phases, since the magnitude of the entropic term in the free energy decreases with temperature, leading to secondary phase growth at intermediate temperatures.^[^ ^19^ ^]^ The 13 nm thick films sputtered at 350 °C were treated with RTA at 600 °C for additional dwell times. Those annealed for a shorter duration of 10 s and 15 s exhibit *fcc* peaks in addition to a minor secondary phase peak near 45° (Figure S2, Supporting Information), which is consistent with the (110) peak of the *bcc* phase seen in the films grown at high temperature. After 20 s of RTA, only the *fcc* phase is detected.

The film surface morphology was probed using scanning electron microscopy (SEM) and atomic force microscopy (AFM). The upper panels of Figure 1b show the SEM images of film surface for increasing T_s_, where a large change in morphology is observed. The films grown at T_s_ up to 350 °C are flat and continuous (not shown). At T_s_ = 500 °C, the film roughness increases, exhibiting a granular structure of particles with an average diameter of ≈20 nm. For T_s_ of 600–700 °C, the films break up into islands, likely due to dewetting, with average size increasing from ≈50 to 80 nm. A similar effect occurs for prolonged RTA times at 600 °C, as illustrated in the SEM images in the lower panels of Figure 1b for 13 nm thick films grown at 350 °C. The film remains smooth and continuous up to 15 s of RTA. After 20 s of RTA, areas of dark contrast emerge, corresponding to void formation in the film which is confirmed by AFM (Figure S3, Supporting Information). After RTA for 30 s, the voids grow and connect to a 45% area fraction. Similar void formation has been reported before during RTA of thin metallic films, attributed to strain relaxation, change in unit cell volume upon phase transformation, and changes in surface energy.^[^ ^15^, ^20^ ^]^ In particular, the high heating rate in RTA has been shown to generate large residual stress in thin films which is relieved upon dewetting.^[^ ^15d^ ^]^ For the 50 nm thick films treated with RTA at 600 °C for 30 s (Figure S3, Supporting Information), the voids have comparable number density but much smaller size, making up <5% area fraction, indicating the suppression of void growth kinetics in the thicker film.

Sample magnetic properties were measured by vibrating sample magnetometry (VSM) and superconducting quantum interference device (SQUID) magnetometry (Table S2, Supporting Information). The films grown at 350 °C exhibit a saturation magnetization *M_s_* = 595 ± 45 emu cm^−3^ (σ_*s*_ = 70 ± 6 emu g^−1^) as‐grown and 590 ± 30 emu cm^−3^ after RTA for 30 s, lying at the upper end of literature values of 20–80 emu g^−1^ for bulk FeCoNiMnCu systems.^[^ ^6^, ^21^ ^]^ The wide range of reported *M_s_* suggests that the stability of long‐range ferromagnetic (FM) order and a large net magnetic moment can be sensitive to small changes of the film composition and the lattice constant.^[^ ^22^ ^]^ Particularly, Mn is expected to reduce the magnetization due to antiferromagnetic (AF) nearest‐neighbor exchange interactions, whereas addition of Cu to FeCoNiMn is suggested to stabilize a FM order.^[^ ^21b^ ^]^ To gauge the sensitivity of *M_s_* on the presence of non‐FM Mn and Cu in the present system, we have performed density functional theory (DFT) calculations of the magnetic moment per Mn atom in Fe_0.19_Co_0.18_Ni_0.20_Mn*_x_* Cu_0.43‐_ *_x_* for different Mn:Cu ratios (Figure S4, Supporting Information). The calculations used the thin‐film lattice constant obtained by XRD, and the compositions used in the calculations are chosen based on the EDX measurement from our samples. The DFT results reveal a FM ground‐state ordering of Mn‐Mn for *x* ≤ 0.25 and AF ordering for *x* > 0.25, suggesting that the net *M_s_* can vary significantly even near the equiatomic composition in our thin film. Also, the maximum magnetic moment of Mn is found close to the experimental Mn:Cu ratio in our films. This suggests that the sizable *M_s_* in our system may be partly attributed to the lower Mn:Cu ratio which promotes FM ordering and high moment of Mn. Further increasing the substrate temperature (T_s_ > 500 °C) leads to an increased *M_s_* likely due to separation of the Fe‐rich phase observed in XRD (Figure 1a), reaching a value of 675 ± 25 emu cm^−3^ at T_s_ = 700 °C.

**Figure 2:**
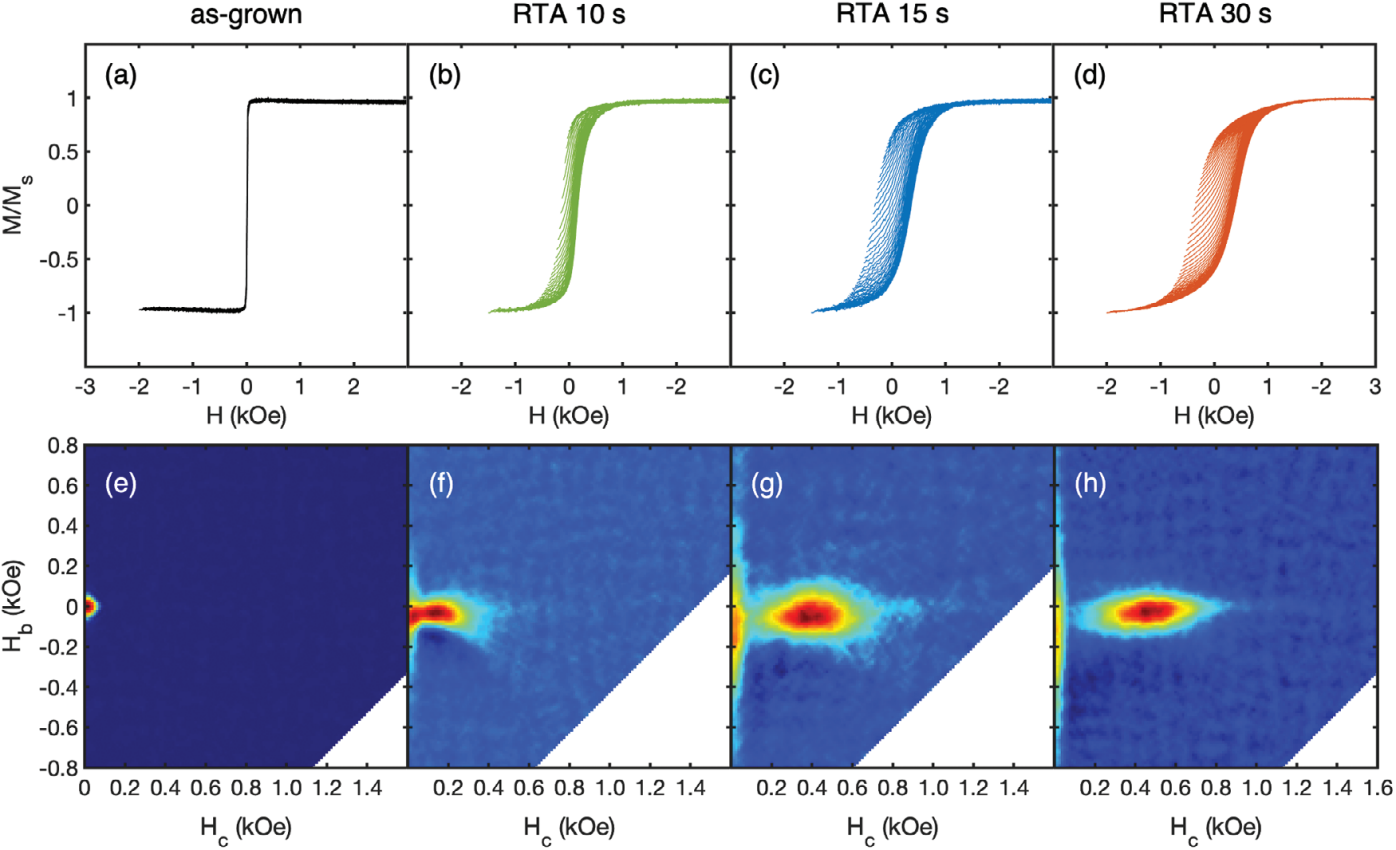
FORC study of 13 nm FeCoNiMnCu films in the in‐plane geometry. a–d) Families of FORCs and e–h) the corresponding FORC distributions (a,e) after sputtering at 350 °C, and after subsequent RTA at 600 °C for b,f) 10 s, c,g) 15 s, and d,h) 30 s.

To better illustrate the evolution of sample magnetic characteristics with changing RTA conditions, we have performed first‐order reversal curve (FORC) analysis in the in‐plane geometry,^[^ ^23^ ^]^ as shown in **Figure** **2**. The as‐grown films sputtered at 350 °C are magnetically soft, with a small coercivity of 10 Oe (Figure 2a), and the FORC distribution exhibits a single peak near local coercivity *H_c_*  = 0, revealing mostly reversible switching (Figure 2e).^[^ ^24^ ^]^ After RTA at 600 °C for 10 s, the sample develops a sizable hysteresis, with a coercivity of 170 Oe (Figure 2b). The FORC distribution shows a separate peak centered around *H_c_* = 150 Oe, indicating irreversible switching in the film (Figure 2f). Additionally, the previous peak near *H_c_* = 0 still persists, but becomes spread along the *H_b_* axis, which indicates reversible magnetization switching due to thermal or anisotropic demagnetization effects.^[^ ^24^, ^25^ ^]^ After 15 s and 30 s of RTA, the samples exhibit an even wider hysteresis with a coercivity of 350 Oe and 390 Oe (Figure 2c,d), respectively. The primary FORC peaks centered at *H_c_* = 400 Oe and 450 Oe, respectively, are clearly distinct from the reversible ridge near *H_c_* = 0, while the latter feature becomes more spread along the *H_b_* axis (Figure 2g,h). By numerical integration of the FORC distribution around the irreversible switching feature, we find that it is associated with 60% of the magnetization in Figure 2h.

The 1–2 orders of magnitude coercivity increase in RTA‐treated samples, compared to the as‐grown ones, suggests the emergence of sizeable magnetic anisotropy in the film. The fact that the continuous 15s RTA sample (Figure 1b) exhibits a 350 Oe coercivity, indicates that the dewetting process and film morphology evolution in some of the samples are not essential to the significant coercivity enhancement. Previous studies on *bulk* FeCoNiMnCu solid solutions have not found appreciable magnetic anisotropy ascribed to the disordered phase.^[^ ^21^, ^26^ ^]^ Hard magnetic properties in some solid solutions have been attributed to the magnetocrystalline or shape anisotropy of a secondary phase, however, studies of bulk phase‐separated FeCoNiMnCu have not shown large coercivity of the order presented here.^[^ ^21a,c^ ^]^ In the present RTA‐treated samples, likely the anisotropic strain has induced lattice and grain distortions or directional chemical order, different from those in bulk systems, and stabilized sizable magnetic anisotropy, similar to that in RTA‐assisted synthesis of *L*1_0_ FeCuPt.^[^ ^15^, ^27^ ^]^

To gain further insight into the coercivity mechanism, angular dependence of the coercivity as a function of RTA time has been measured and well‐fit to a model based on domain wall displacement (Figure S5, Supporting Information), indicating a pinning‐controlled coercivity for the in‐plane direction.^[^ ^28^ ^]^ The evolution of fitting parameters also reveals significant deviations in the overall demagnetization factors from the thin film geometry after RTA, even before film dewetting, reflecting the change in anisotropy induced by RTA. Therefore, the hard magnetic properties in FeCoNiMnCu film may be explained by RTA‐induced microstructure changes and anisotropy enhancement which strengthens domain wall pinning.

While the combination of saturation magnetization and coercivity for these RTA‐ treated FeCoNiMnCu films is impressive in comparison to previously reported bulk systems, the coercivity is still modest, due to the high symmetry of the cubic lattice which limits the intrinsic magnetocrystalline anisotropy. The facilitation of low‐symmetry chemical order and crystal structure combined with strong spin‐orbit coupling is in general necessary to realize strong magnetocrysalline anisotropy. To achieve these conditions in a high entropy system, we have added 50 at.% Pt to the FeCoNiMnCu alloy, employing the high entropy intermetallic design approach in search of highly anisotropic *L*1_0_ ordering. Although the certainty of Pt sites reduces the configurational entropy compared to the equiatomic case, we demonstrate below that such optimized entropy can still stabilize a single high‐entropy *L*1_0_ phase.

**Figure 3:**
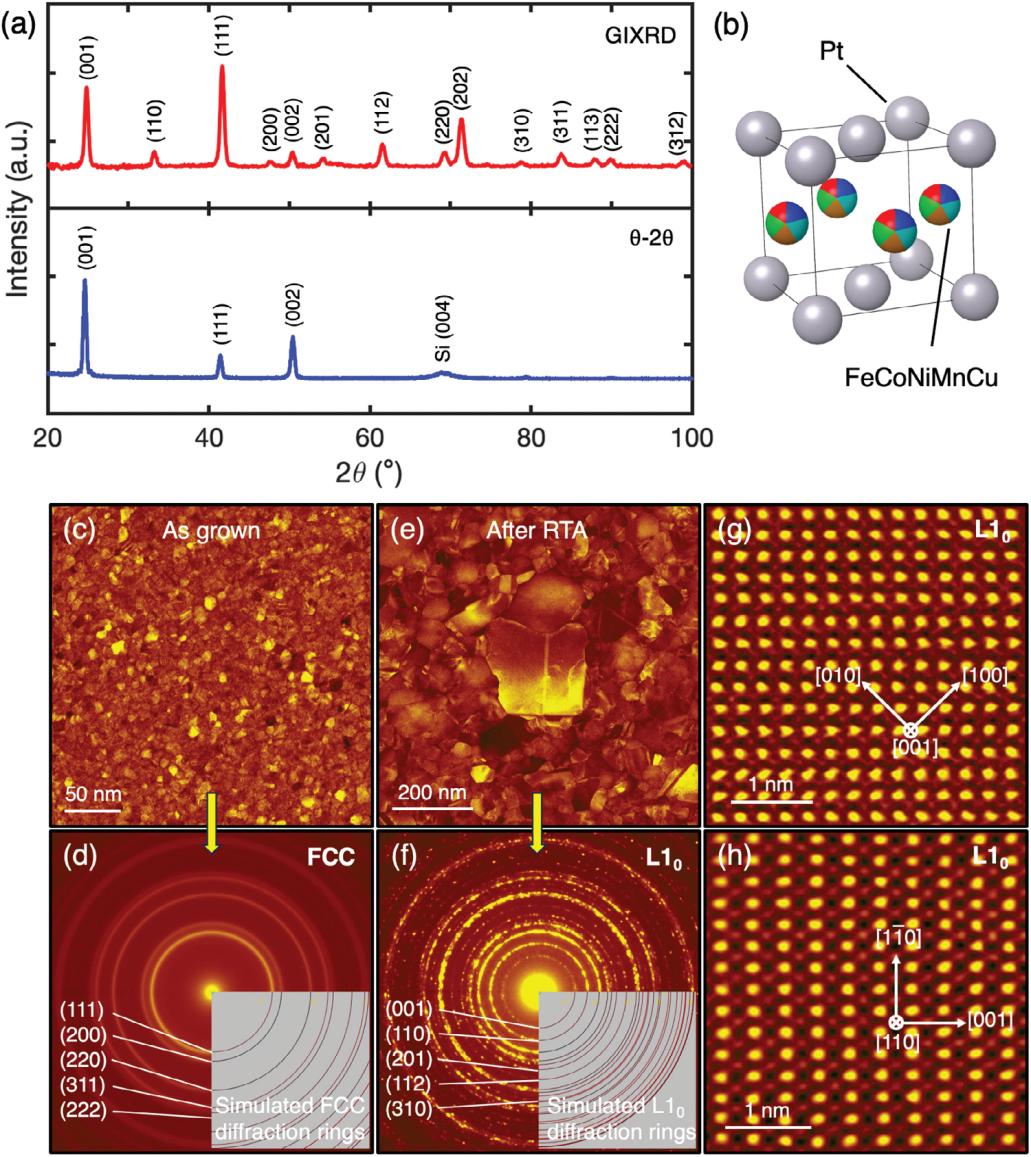
XRD and plan‐view STEM microstructural study of (FeCoNiMnCu)Pt films. a) (Top) GIXRD and (bottom) ***θ***‐2 ***θ*** XRD scans for (FeCoNiMnCu)Pt films after RTA at 600 °C for 60 s. b) Schematic crystal structure of *L*1_0_ (FeCoNiMnCu)Pt. c) HAADF‐STEM image showing uniform nano‐grains from the as grown sample, and d) the corresponding SAED pattern showing a well‐defined fcc structure. e) HAADF‐STEM image showing grown grains from the RTA sample, and f) the corresponding SAED pattern showing a well‐defined *L*1_0_ structure with indexing of selected forbidden fcc reflections. g,h) Atomic resolution HAADF‐STEM images taken along the [001] and [110] zone‐axis, respectively, showing the chemical ordering of the *L*1_0_ structure on the FeCoNiMnCu and Pt sites.

Thin films of 20 nm thick (FeCoNiMnCu)Pt were sputtered as described in the Experimental Section. EDX analysis revealed an average film composition of Fe_0.11_Co_0.12_Ni_0.10_Mn_0.09_Cu_0.10_Pt_0.48._ The as‐grown films are in a disordered *fcc* (A1) phase with a lattice parameter of 3.80 Å (not shown). After RTA for 60 s at 600 °C, *L*1_0_ ordering appears in GIXRD and θ‐2θ (1° ω‐offset) scans (**Figure** **3**a), evidenced by the emergence of the forbidden (001) superlattice peak and peak‐splitting due to the tetragonal symmetry. The extracted lattice parameters of the *L*1_0_ phase are *c* = 3.60 Å and *a =* 3.83 Å, respectively. The presence of *L*1_0_ peaks in the GIXRD scan shows the existence of randomly oriented *L*1_0_ grains. However, the θ‐2θ XRD reveal dominant (00*l*) reflections, with a narrow (001) rocking‐curve (full‐width‐at‐half‐maximum of 2°), indicating a preferred (001) orientation. The *L*1_0_ order parameter can be extracted from the ratio of integrated intensities of the superlattice and fundamental peaks according to $S\;=\sqrt{\frac{{\left(I_{001}/I_{002}\right)}_{exp.}}{{\left(I_{001}/I_{002}\right)}_{calc.}}}\;$ where the numerator includes the (001) and (002) intensities extracted from the experimental θ‐2θ XRD data, and the denominator includes the calculated intensities.^[^ ^29^ ^]^ A high *S*‐value of 0.8 is found, indicating the presence of significant *L*1_0_ ordering, illustrated in Figure 3b.

High angle annular dark field scanning transmission electron microscopy (HAADF‐STEM) and selected area electron diffraction (SAED) were further carried out to characterize the microstructures of the (FeCoNiMnCu)Pt films. As shown in Figure 3c, HAADF‐STEM imaging on the as‐grown (FeCoNiMnCu)Pt film shows a uniform grain size ≈5–10 nm. SAED analysis (Figure 3d) shows that the as‐grown film was crystallized into a well‐defined *fcc* structure. After RTA processing, the grains grew substantially from several tens of nm up to 300 nm and transformed into *L*1_0_ structure, indicated in SAED by the appearance of additional reflections forbidden for the *fcc* phase, such as the (001), (110), (201), (112) and (310) reflections (Figure 3e,f). The large grain growth relative to some RTA‐treated FePt films may be related to the inclusion of Cu which is known to promote diffusion and reduce the kinetic ordering temperature.^[^ ^15^, ^30^ ^]^ Atomic resolution HAADF‐STEM images taken along the [001] and [110] zone‐axis not only reveal the fourfold and twofold symmetry, respectively, but also illustrate the striking chemical ordering of the *L*1_0_ phase, apparent by the Z‐contrast between FeCoNiMnCu and Pt sites (Figure 3g,h). Further EDX analysis was conducted on ≈50 grains and found no significant composition variations between grains or existence of binary phases. Therefore, we consider the film to be a single‐phase high entropy intermetallic, with the 3*d* transition metals ordering onto a high entropy sublattice of the *L*1_0_ structure, opposite to Pt.

**Figure 4:**
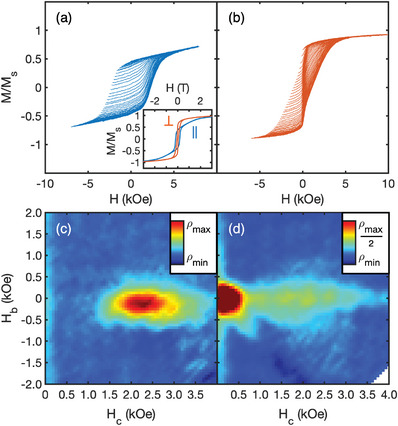
FORC of (FeCoNiMnCu)Pt film after RTA. a) In‐plane and b) out‐of‐plane family of FORCs and c,d) corresponding FORC distributions for (FeCoNiMnCu)Pt films after RTA at 600 °C for 60 s. Note the adjustment of color scale in (d). Inset in (a) shows the major loop in the in‐plane (blue) and out‐of‐plane (brown) geometry.

Magnetic properties of the (FeCoNiMnCu)Pt films have been studied by magnetometry and FORC. The as‐grown films are magnetically soft with in‐plane anisotropy and coercivity of 5 Oe (not shown). After RTA for 60 s at 600 °C, strong magnetic anisotropy has emerged, as shown by the families of FORCs and the corresponding FORC distributions in **Figure** **4**. Large coercivity increases to 2.50 kOe and 1.46 kOe are observed for the in‐plane (IP) and out‐of‐plane (OOP) loop (Figure 4a inset), respectively, which are also delineated by the outer boundaries of the FORCs (Figure 4a,b). The high *M_r_*/*M_s_* in the OOP loop relative to IP indicates the perpendicular anisotropy resulting from the (001) texture, as shown by the inset to Figure 4a. For the IP geometry, significant irreversible switching is revealed by the FORC peak centered at *H_c_* = 2.2 kOe, arising from high anisotropy *L*1_0_ grains with IP easy‐axis component (Figure 4c). Integrating this feature shows that it corresponds to 45% of the magnetization, while the remaining magnetization exists in the reversible ridge, representing magnetization reversal along the hard‐axis of (001)‐textured grains. For the OOP geometry, the FORC distribution exhibits two main features: a sharp peak centered at *H_c_* = 0.2 kOe and a broader horizontal ridge along the *H_c_* axis centered at *H_c_* = 2.0 kOe (Figure 4d). The FORC feature near the origin is notably different from a typical low anisotropy phase, which may be manifested as a vertical ridge along the *H_b_* axis due to shape anisotropy, as seen in the OOP FORC distribution of FeCuPt thin films containing the *A*1 phase.^[^ ^24b^ ^]^ Here, the low field FORC feature corresponds to a square loop contribution in the OOP direction (Figure 4c) despite the shape anisotropy energy, suggesting that it stems from a phase of sizeable perpendicular magnetic anisotropy. This feature likely originates from the large *L*1_0_ grains seen in STEM (Figure 3e) which exhibit lower coercivity due to the absence of strong pinning centers such as grain boundaries and higher probability of surface nucleation sites. For example, bulk single‐crystal *L*1_0_ FePt may exhibit negligible coercivity despite its enormous anisotropy, due to the ease of domain wall motion through bulk single‐crystals.^[^ ^31^ ^]^ Meanwhile, grains with smaller sizes or varying *c*‐axis orientation promote hysteresis through pinning at their grain boundaries. Integration of the OOP FORC feature at higher *H_c_* yields 45% of the saturation magnetization, equivalent to the primary FORC feature in the IP geometry. We propose that these high *H_c_* FORC features are associated with small and randomly oriented grains, which are evident by the observation of *L*1_0_ peaks in GIXRD (Figure 3a). Therefore, the bimodal coercivity distribution observed in the OOP direction is explained by a 2‐step process of magnetization reversal beginning with easy domain nucleation and propagation in the large (001) oriented grains (narrow low *H_c_* peak), followed by pinning between grains with small size and varying easy‐axis direction (broad high *H_c_* peak). The distribution of size and easy‐axis orientation both manifest in the spreading of the high *H_c_* peak along the *H_c_* axis. Based on this, we expect that the *H_c_* could be enhanced through microstructural optimization such as a reduction in grain size, which may be achieved by adjusting the RTA time and temperature.

An effective uniaxial anisotropy constant of *K_u_* = 2 × 10^6^ erg cm^−3^ was extracted from the area between IP and OOP hysteresis loops after correction of a shape component 2π*M_s_* ^2^. While not representative of the intrinsic magnetocrystalline anisotropy due to the polycrystalline microstructure, we note that *K_u_* extracted here is already in the range of moderate high magnetic anisotropy materials, although still somewhat lower than the intrinsic anisotropy of the binary *L*1_0_ FePt and CoPt alloys, which are on the order of 10^7^ erg cm^−3^. The saturation magnetization *M_s_* of 240 ± 25 emu cm^−3^ for the Pt‐inserted film, which is roughly half of the *M_s_* obtained for the FeCoNiMnCu films, is reduced in comparison to the bulk values of FePt and CoPt. The *K_u_* of Pt‐based *L*1_0_ phases are determined to first‐order by the effective VEC of the 3*d* metal sublattice (i.e., Fermi level).^[^ ^32^ ^]^ Based on the experimental composition, the reported *L*1_0_ HEA films have an effective VEC of 9.0, and their *K_u_* is reasonably consistent with that of *L*1_0_‐ordered (Fe‐Co‐Ni)Pt films having VEC of 9.1, which exhibit *K_u_* on the order of 10^6^ erg cm^−3^.^[^ ^33^ ^]^

## 3 Conclusion

In summary, we have achieved high magnetic anisotropy in rare‐earth‐free high entropy metallic thin films by sputtering and RTA. Robust magnetic order of both *fcc* FeCoNiMnCu and *L*1_0_ (FeCoNiMnCu)Pt films has been demonstrated. The FeCoNiMnCu films exhibit a large coercivity increase after RTA, resulting from microstructural changes and strain‐induced anisotropy enhancement which strengthens domain wall pinning. Addition of Pt reduces the symmetry of the cubic *fcc* lattice to an intermetallic tetragonal *L*1_0_ phase of (FeCoNiMnCu)Pt. Although the Pt insertion lowers the configurational entropy compared to the equiatomic case, the entropy is still high enough to stabilize an *L*1_0_ single phase. This intermetallic phase is confirmed to host high magnetocrystalline anisotropy, likely attributed to the lower symmetry of the *L*1_0_ structure and the strong spin‐orbit coupling provided by the Pt insertion. These findings demonstrate the promise of high‐entropy intermetallic design as a key avenue to search for new high‐entropy magnets beyond the equiatomic composition.

## 4 Experimental Section

The design of these HEA thin films is based on the estimation of mixing enthalpy ΔH_mix_ using the Miedema method.^[^ ^34^ ^]^ A table of binary ΔH_mix_ for 3*d* metals is given in Table S1 (Supporting Information).^[^ ^16^ ^]^ The element‐weighted average mixing enthalpy of the solid solution FeCoNiMnCu is Δ H_mix_ = 1.23 kJ mol^−1^. The small positive enthalpy was outweighed by the ideal configurational entropic contribution to the free energy at room temperature, − T S_config_ = − 3.9 kJ mol^−1^. The valence electron concentration of 9 for equiatomic FeCoNiMnCu would correspond to a *fcc* structure for 3*d* transition metal HEAs,^[^ ^18^ ^]^ which is a precursor to the *L*1_0_ structure. The system (FeCoNiMnCu)Pt also satisfied the geometric and electronic criteria for single‐phase HEIs with atomic size difference δ*r*  = 5.7% and modified electronegativity difference η  = 0.27.^[^ ^12b^ ^]^

The FeCoNiMnCu films were deposited by direct‐current magnetron sputtering in an ultrahigh vacuum system with a base pressure of ∼1 × 10^−8^ Torr. A homemade composite Fe‐Co‐Ni‐Mn‐Cu target was used, which was formed by cold‐pressing a mixture of elemental powders, with purities of 99.9% (Fe), 99.8% (Co), 99.9% (Ni), 99.6% (Mn), and 99.9% (Cu). The particle size of all powders was under 10 µm. The powders were mixed in an equimolar ratio, and the mixture was uniaxially pressed with 50 metric tons onto a Cu backing plate, forming a target 2″ in diameter and 1/8″ thick. Films of 13 and 50 nm thickness were sputtered in 2.5 mTorr Ar with 50 W power onto thermally oxidized Si (100) substrates with a 285 nm thick amorphous SiO_2_ layer. Substrate temperature was held at T_s_ = 20, 350, 500, 600, and 700 °C. The films were cooled in 30 mTorr of Ar for up to 1 h before deposition of 4–5 nm of Ta or Ti capping layer to prevent oxidation. The (FeCoNiMnCu)Pt films of 20 nm thickness were deposited onto thermally oxidized Si (100) substrates at room temperature in 2 mTorr Ar, by co‐sputtering of elemental Fe, Co, Ni, Mn, Cu, and Pt targets using 35, 30, 28, 20, 13, and 70 W power, respectively.^[^ ^35^ ^]^ Additional 20 nm (FeCoNiMnCu)Pt films were deposited onto Si/SiO_2_ by co‐sputtering of Pt with the composite FeCoNiMnCu target in 2 mTorr Ar at powers of 20 and 70 W, respectively. The results are reported in Figures S6 and S7 (Supporting Information).

Sputtered films were treated with RTA in a vacuum chamber with a base pressure ∼1 × 10^−8^ local coercivity Torr. During RTA, the sample was transferred from an adjacent load‐lock chamber under vacuum into the main chamber where it was placed directly under a heating lamp for the designated annealing time, ranging from 10 to 60 s. The annealing temperature of 600 °C corresponded to the equilibrium temperature of the surrounding chamber environment, as set via thermocouple in contact with the lamp window, located ≈1–2 cm from both the sample and heating filament. Subsequently, the sample was transferred back to the load‐lock, which was vented with dry N_2_ and brought to atmospheric pressure over a period of ≈2 min, during which the sample was cooled to room‐temperature.

X‐ray diffraction with Cu K_α_ radiation was employed for structural characterization using a Panalytical X'Pert3 Materials Research Diffractometer. Surface topography was imaged on Zeiss SUPRA 55‐VP scanning electron microscope and NT‐MDT atomic force microscope. An Oxford Instruments EDX system was used to analyze chemical composition of the films. High‐angle annular dark field scanning transmission electron microscopy (HAADF‐STEM) and selected area electron diffraction (SAED) were performed on plan‐view samples for microstructural analysis.

Magnetic measurements were performed via vibrating sample magnetometry (VSM) using a Princeton Measurements Corporation MicroMag and superconducting quantum interference device (SQUID) magnetometry on a Quantum Design Magnetic Property Measurements System (MPMS3) system. FORC measurements were carried out following prior procedures.^[^ ^20^ ^]^ The sample was first saturated, then brought to a reversal field (*H_R_*), and its magnetization *M*(*H*, *H_R_*) measured in an applied field *H* back to saturation. This process was repeated at successively more negative *H_R_* to create a family of FORCs. A FORC distribution was then extracted using $\rho \;\left(H,H_{R}\right)\equiv -\frac{1}{2M_{S}}\frac{\partial^{2}M\left(H,H_{R}\right)}{\partial H\;\partial H_{R}},$ where *M_S_* is the saturation magnetization. The FORC distribution is represented in terms of local coercive field $H_{c}=\frac{1}{2}\;\left(H-H_{R}\right)$ and bias field $H_{b}=\frac{1}{2}\;\left(H+H_{R}\right).$ ^[^ ^36^ ^]^ The local coercivity often correlates with, but is not necessarily the same as, the major loop coercivity.

First‐principles calculations were carried out for bulk HEAs corresponding to the compositions and the lattice constants obtained in experiment. The study used density functional theory implemented in Questaal^[^ ^37^ ^]^ with the Korringa–Kohn–Rostocker (KKR) method^[^ ^38^ ^]^ and Coherent‐Potential Approximation (CPA).^[^ ^39^ ^]^ Each high‐entropy species was assumed to have two spin components that were allowed to have either ferro‐ or antiferromagnetic order during the self‐consistent iterations.

### Statistical Analysis

Information of the FeCoNiMnCu films studied is provided in Table S2 (Supporting Information). Saturation magnetization values determined are represented in the form of mean ± standard deviation. Other experimentally measured values are accurate to the last significant digit.

## Conflict of Interest

W.B.B. and K.L. are co‐inventors on a pending patent application on Boron‐based and high entropy magnetic materials filed by Georgetown University.

## Supporting information

Supporting Information

## Data Availability

The data that support the findings of this study are available from the corresponding author upon reasonable request.
