# Supplementary material for: Single‐Phase L10‐Ordered High Entropy Thin Films with High Magnetic Anisotropy
Source: Adv Sci (Weinh). 2024 Jun 28;11(34):2308574. doi: 10.1002/advs.202308574 (PMC11426066; doi:10.1002/advs.202308574)
Supplement: Supplementary file 1 — Supporting Information [file ADVS-11-2308574-s001.docx]

**Supporting Information**

**Single-Phase *L*1_0_-Ordered High Entropy Thin Films with High Magnetic Anisotropy**

Willie B. Beeson,^1^ Dinesh Bista,^1^ Huairuo Zhang,^2,3^ Sergiy Krylyuk,^2^ Albert Davydov,^2^ Gen Yin,^1^ and Kai Liu^1,*^

^1^Department of Physics, Georgetown University, Washington, DC 20057, USA

^2^Materials Science and Engineering Division, National Institute of Standards and Technology, Gaithersburg, MD 20899, USA

^3^Theiss Research, Inc., La Jolla, CA 92037, USA

**Table S1**. Binary $\Delta H_{mix}$ for 3*d* metals.^[1]^ The red lines delineate the Fe-Co-Ni-Mn-Cu system.


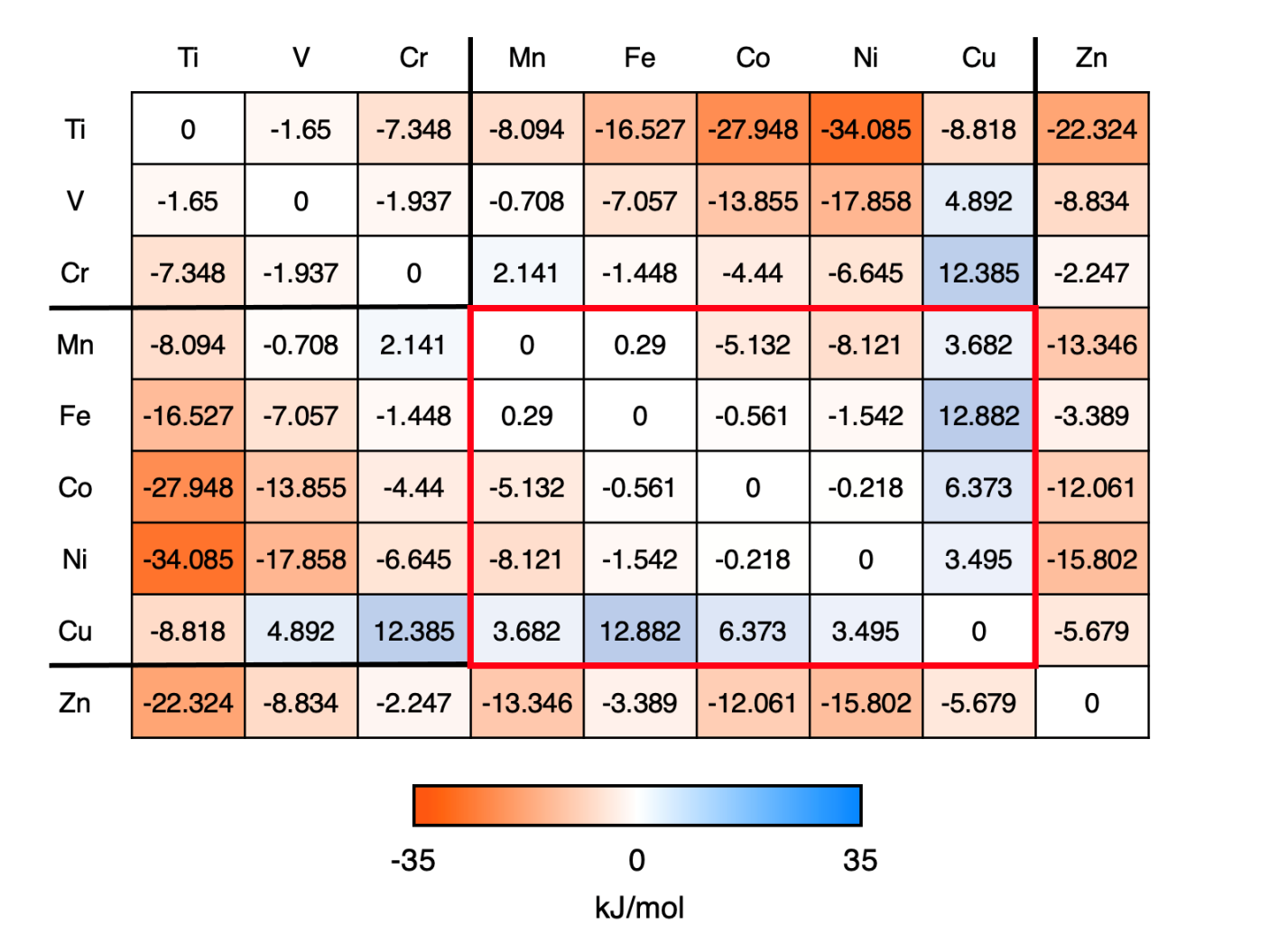


**Table S2**. Fabrication conditions and magnetic properties of FeCoNiMnCu films.


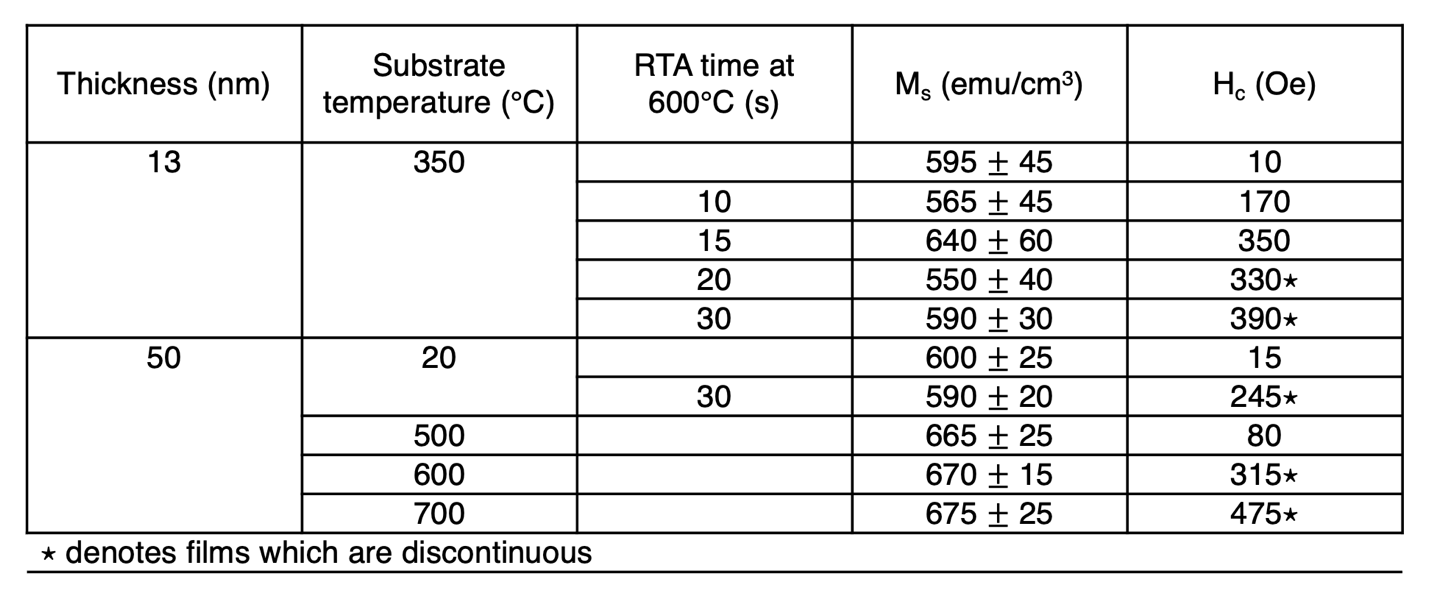


Table S2 lists the studied FeCoNiMnCu film fabrication conditions and magnetic properties. A star is added to coercivity values to denote samples that are not continuous films, due to dewetting which produces either voids or isolated islands. The 50 nm films sputtered at elevated temperature exhibit a large coercivity increase, however this is clearly seen to correlate with the morphology change from thin films to isolated nanoislands, as shown in Fig. 1b, which leads to coercivity enhancement via size effects. Meanwhile, the 13 nm films annealed for 10 s and 15 s exhibit comparable coercivity increase while remaining as continuous thin films.


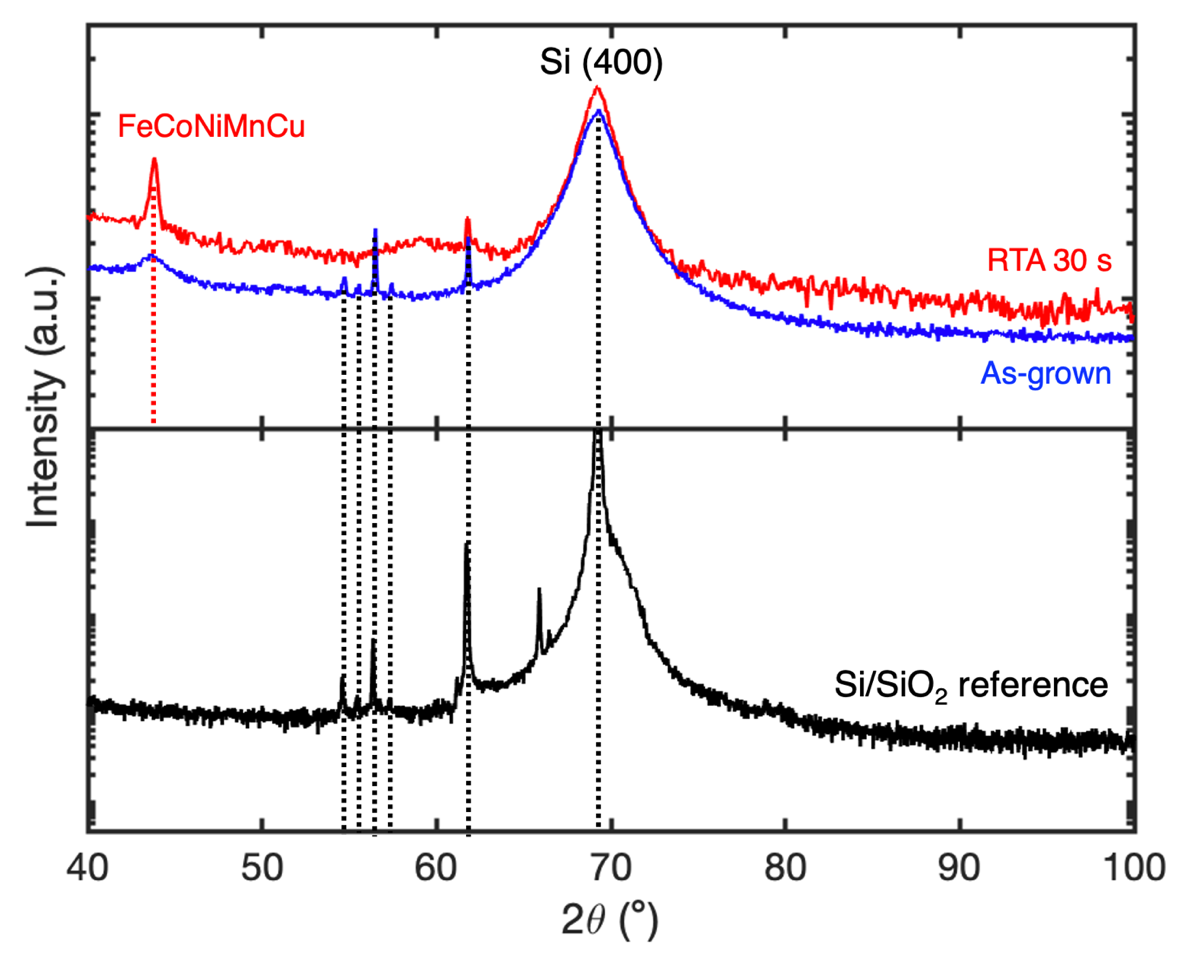


**Figure S1.** (Top) $\theta$-2$\theta$ XRD scan with 1$^{\circ}$ $\omega$ offset of 50 nm FeCoNiMnCu film grown at 20 $^{\circ}$C and after RTA at 600 $^{\circ}$C for 30 s. (Bottom) Reference $\theta$-2$\theta$ scan of Si/SiO_2_ substrate with 0$^{\circ} \omega$ offset.

$\boldsymbol{\theta}$**-2**$\boldsymbol{\theta}$ **XRD of FeCoNiMnCu films**

$\theta$-2$\theta$ XRD scans with $\omega$ offset of 1$^{\circ}$ was performed for the 50 nm FeCoNiMnCu films sputtered at 20 $^{\circ}$C and treated with RTA for 30 s, as seen in the upper panel of Figure S1. Significant crystal growth after RTA can be seen by the increased intensity and narrow width of the primary peak at 44$^{\circ}$. The film after RTA appears to show a preferred (111) texture, due to the lack of significant *fcc* peaks at higher 2$\theta$. The lower panel of Figure S1 shows a reference $\theta$-2$\theta$ scan of the Si/SiO_2_ substrate, accounting for the peaks at 55$^{\circ}$ and above. Note that no $\omega$ offset is used in the reference scan, leading to sharp Si (400) diffraction peaks for Cu K_α_, Cu K_β_, and W L_α_ wavelengths which are absent in the upper panel.


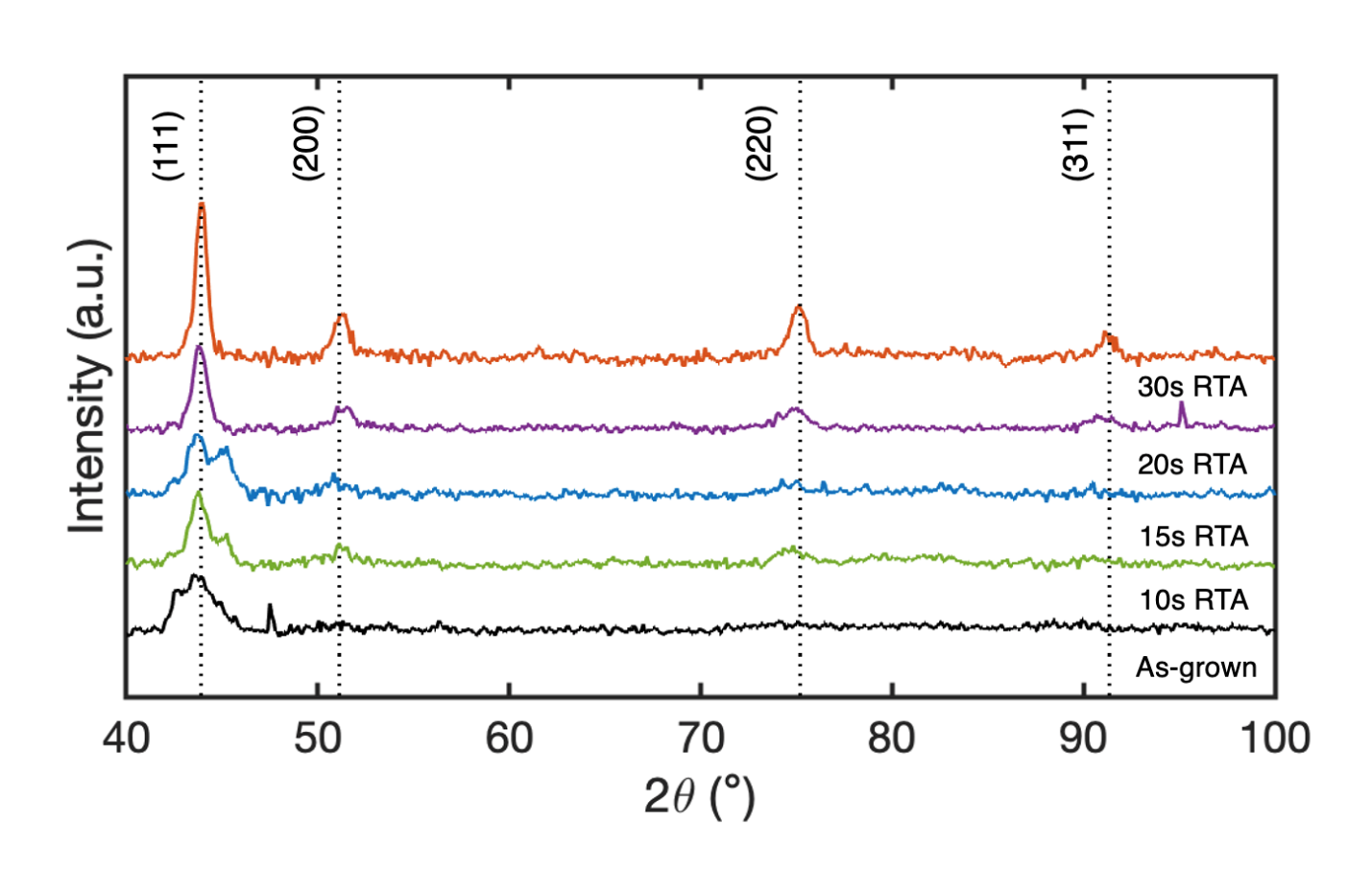


**Figure S2.** GIXRD scans ($\omega=$ 0.4$^{\circ}$) for 13 nm FeCoNiMnCu films sputtered at 350 $^{\circ}$C and after RTA treatment at 600 $^{\circ}$C for 10 s, 15 s, 20 s, and 30 s.

**GIXRD of 13 nm FeCoNiMnCu for different annealing times**

Figure S2 shows the GIXRD scans ($\omega=$ 0.4$^{\circ}$) for the 13 nm FeCoNiMnCu films grown at 350 $^{\circ}$C and treated with RTA for different times. The films treated with RTA were capped with 4 nm Ti layer. The as-grown film shows a broad primary peak at 2$\theta$ = 44$^{\circ}$, similar to the films grown at 20 $^{\circ}$C. The films treated with RTA for 10 s and 15 s exhibit *fcc* peaks in addition to a peak at $\sim$45$^{\circ}$, which is consistent in position with the (110) peak of the *bcc* phase observed in the 50 nm films grown at high temperature (Figure 1). The films annealed for 20 s and 30 s show only *fcc* phase peaks.


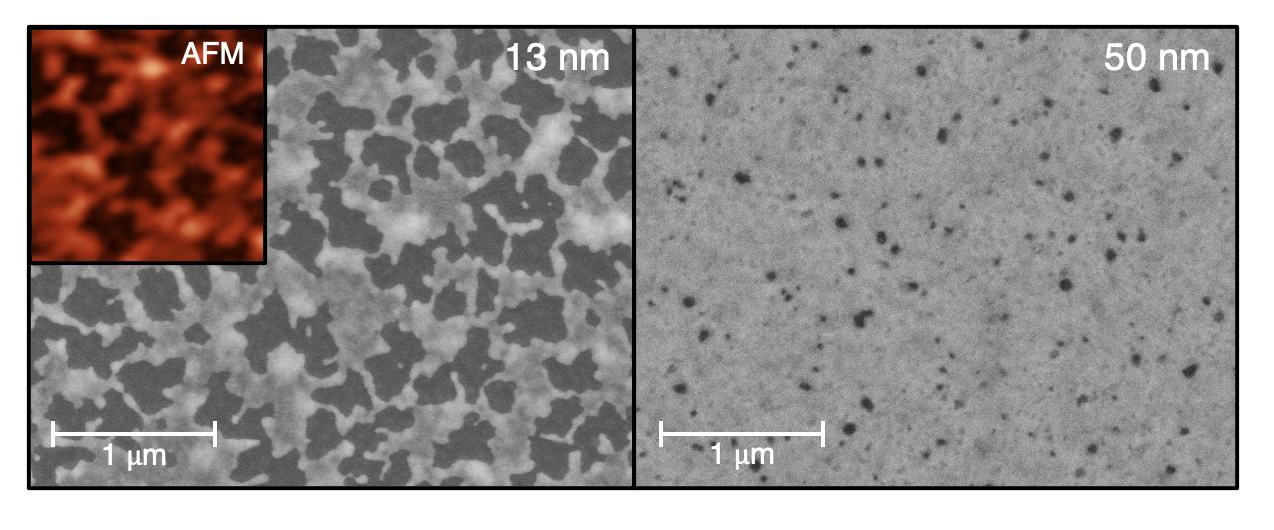


**Figure S3.** SEM images of FeCoNiMnCu films after 30 s of RTA for thicknesses of 13 nm (left) and 50 nm (right). The inset on the left is the AFM measurement of the film surface, showing the height difference corresponding to SEM contrast. The scale bar of the AFM image is the same as the SEM.

**Effect of film thickness on void growth**

SEM images taken after 30 s RTA time reveal the difference in void growth between 13 nm and 50 nm film. The inset for the SEM image of 13 nm film (left) shows the AFM measurement of the sample surface, where the height variation can be seen to match the contrast in SEM. ImageJ software was used to analyze the density and area fraction of voids. In the 13 nm film, voids make up 45% area fraction and have a number density of approximately 5 $\mu$m^-2^, while the voids in 50 nm film make up less than 5% area fraction but with a comparable number density of approximately 8 $\mu$m^-2^. The difference in number density could be attributed to the merging of voids upon growth. The comparable number density but smaller area fraction reflects the vastly suppressed void growth kinetics in the thicker film, while the void nucleation rate is unchanged.


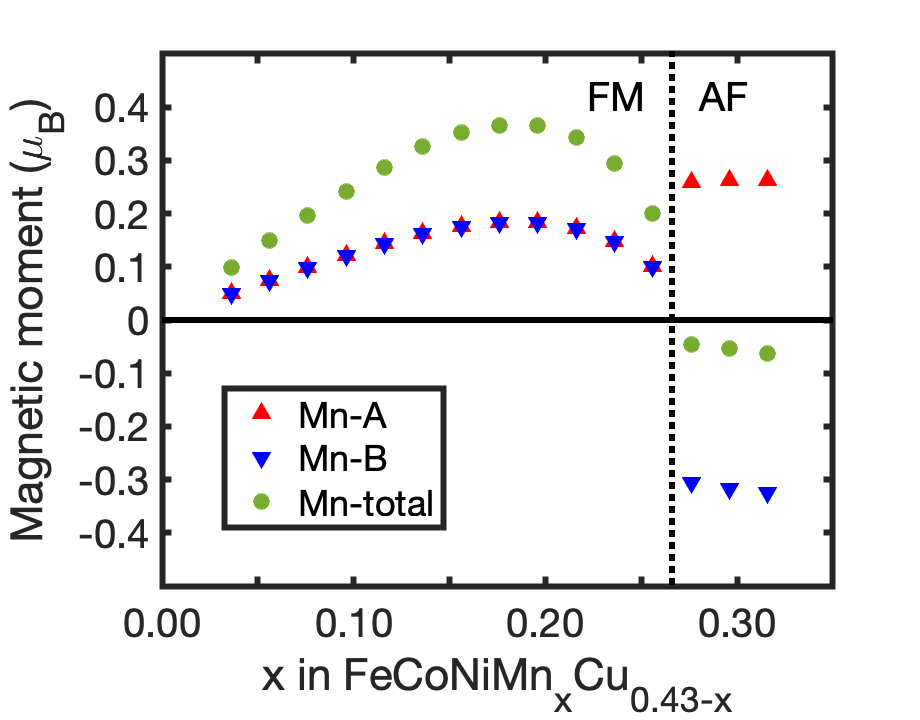


**Figure S4.** Calculated magnetic moments of Mn in Fe_0.19_Co_0.18_Ni_0.20_Mn_x_Cu_0.43-x_ given by DFT. The magnetic configuration of the random Mn sites is explored by allowing 50% of Mn sites (Mn-A, red up triangle) to host either FM or AF moments compared to the other 50% (Mn-B, blue down triangle). Mn-total (green circle) represents the sum of moments for Mn-A and Mn-B at the ground state.

**Calculated magnetic moment of Mn in Fe_0.19_Co_0.18_Ni_0.20_Mn_x_Cu_0.43-x_.**

We have used density functional theory (DFT) to understand the ground-state magnetization configuration for the near equiatomic Fe_0.19_Co_0.18_Ni_0.20_Mn_x_Cu_0.43-x_. We have employed the DFT implemented in Questaal using the Korringa-Kohn-Rostocker (KKR) method and the mean-field Coherent-Potential Approximation (CPA). In this calculation we allow each high-entropy element to have half of random sites being AF or FM aligned with the other half, and search for a lower ground-state energy. As shown in Figure S4, a FM to AF transition for Mn sites is found when changing the concentration ratio of Mn:Cu (Figure S4). All other elements, however, are found to have pure FM phase regardless of the Mn concentration.


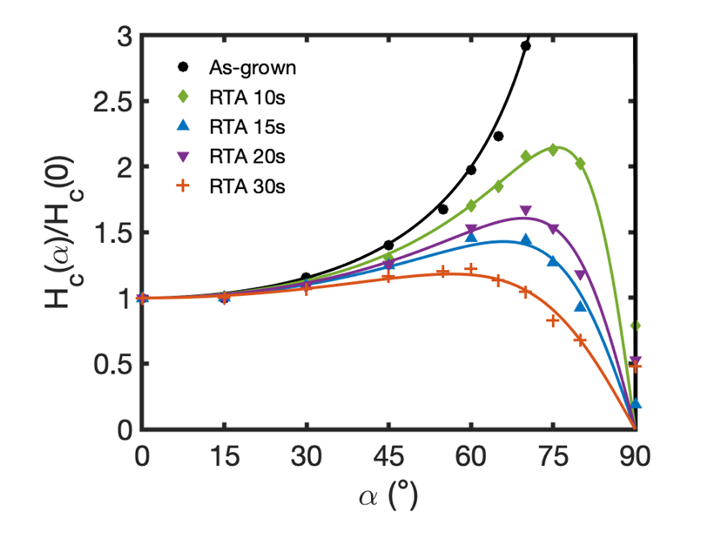


**Figure S5.** Dependence of coercivity on the angle 𝛼 between 𝐻 and the film plane for the FeCoNiMnCu films after RTA for different times. The fits of the data to Equation 1 are shown by the solid lines.

**Angular dependence of coercivity**

The angular variation in coercivity was measured to gain further insight into the evolution of magnetic properties. Figure S5 shows the dependence of the normalized coercivity $H_{c}(\alpha)/H_{c}(0)$ on the angle $\alpha$ between the field direction and film surface. $H_{c}(0)$ is the coercivity measured in-plane. The angular variation in coercivity for multi-domain crystals due to the combination of domain wall motion and coherent reversal may be described by a generalized Kondorsky model:^[2]^

$\frac{H_{c}\left( \alpha\right)}{H_{c}\left( 0 \right)}=\frac{\cos\alpha}{{N\sin}^{2} \alpha+\cos^{2} \alpha}$ (1)

where $N$ is a parameter relating the demagnetization factors as $N=\frac{N_{x}}{N_{z}+N_{A}}$, with $N_{z}$ and $N_{x}=N_{y}$ being the demagnetization factors in the out-of-plane ($\alpha=$ 90$^{\circ}$) and in-plane ($\alpha=$ 0$^{\circ}$) directions, respectively, and more generally the $\alpha=$ 0$^{\circ}$ defines the easy-axis; $N_{A}$ represents an effective demagnetization factor due to uniaxial anisotropies other than the shape. The equation reduces to the Kondorsky relation $\left( \cos\alpha\right)^{-1}$ for $N_{x}=$ 0. The model predicts $H_{c}\left( 90^{\circ} \right)=$ 0 for perfect single-crystals, whereas $H_{c}\left( 90^{\circ} \right)\neq$ 0 for polycrystals. Excluding $H_{c}\left( 90^{\circ} \right)$, the data is reasonably well-fit to Equation 1, which indicates the pinning-controlled coercivity mechanism for angles close to in-plane. The deviation from $H_{c}$ $\propto\left( \cos\alpha\right)^{-1}$ at higher angles signals a transition to coherent rotation or nucleation-controlled coercivity. In the as-grown state, $N$ = 0.0007, reflecting the extreme thin film shape demagnetization factors, while RTA treatment for 15 s leads to an increase of $N$ to 0.143. After 20 s of RTA, there is a slight decrease to 0.108 then finally an increase to 0.233 after 30 s of RTA. Here, an increase in $N$ effectively indicates the reduction of in-plane anisotropy. This could relate to shape anisotropy changes as a result of dewetting in the case of the films annealed for 20 s and 30 s. However, a substantial change of $N$ is observed after 15 s RTA without any obvious change in film morphology. In this case, the increase of $N$ could reflect local changes in shape anisotropy or other forms of strain-induced anisotropy leading to a negative value of $N_{A}$. Notably, upon film dewetting at 20 s RTA, there is a slight decrease of $N$, while the morphology changes alone would be expected to increase $N$. Considering the observed reduction of residual stress upon film dewetting in similar RTA-treated films,^[3]^ this behavior is consistent with the proposed role of strain on the modification of anisotropy in the present films. The overall trend observed here illuminates the formation of the reversible ridge in the in-plane FORC distributions due to an increase of demagnetization effects in the film plane.


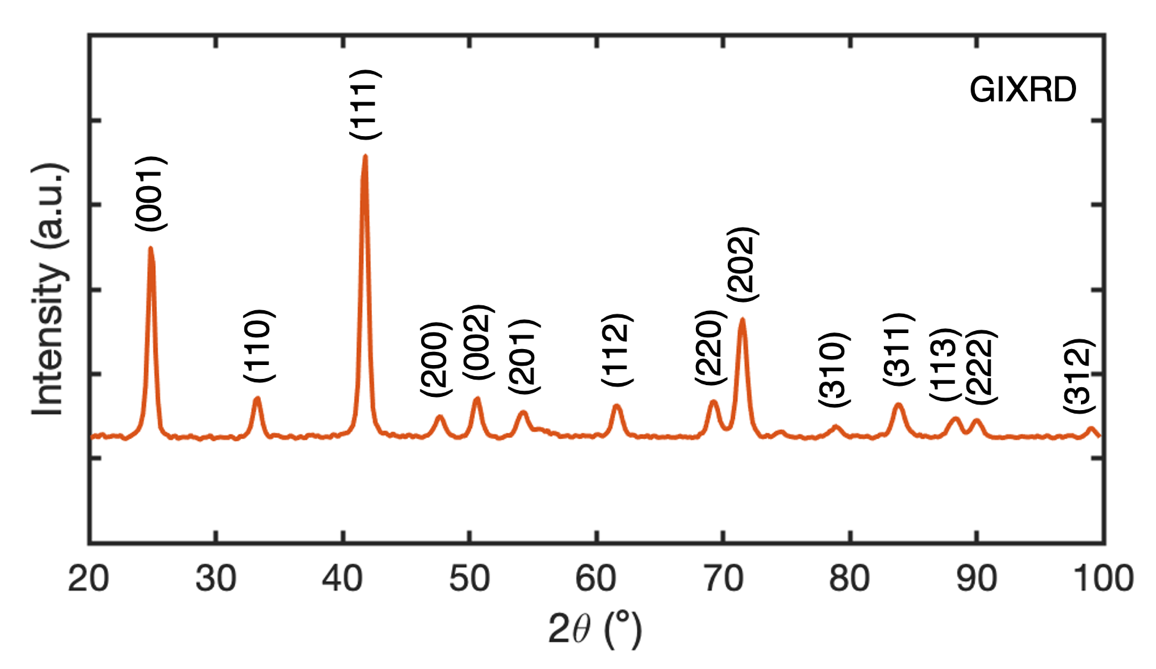


**Figure S6.** GIXRD scans ($\omega=$ 0.4$^{\circ}$) of 20 nm (FeCoNiMnCu)Pt films deposited by co-sputtering of the composite FeCoNiMnCu and elemental Pt targets and treated with RTA at 600 °C for 60 s.


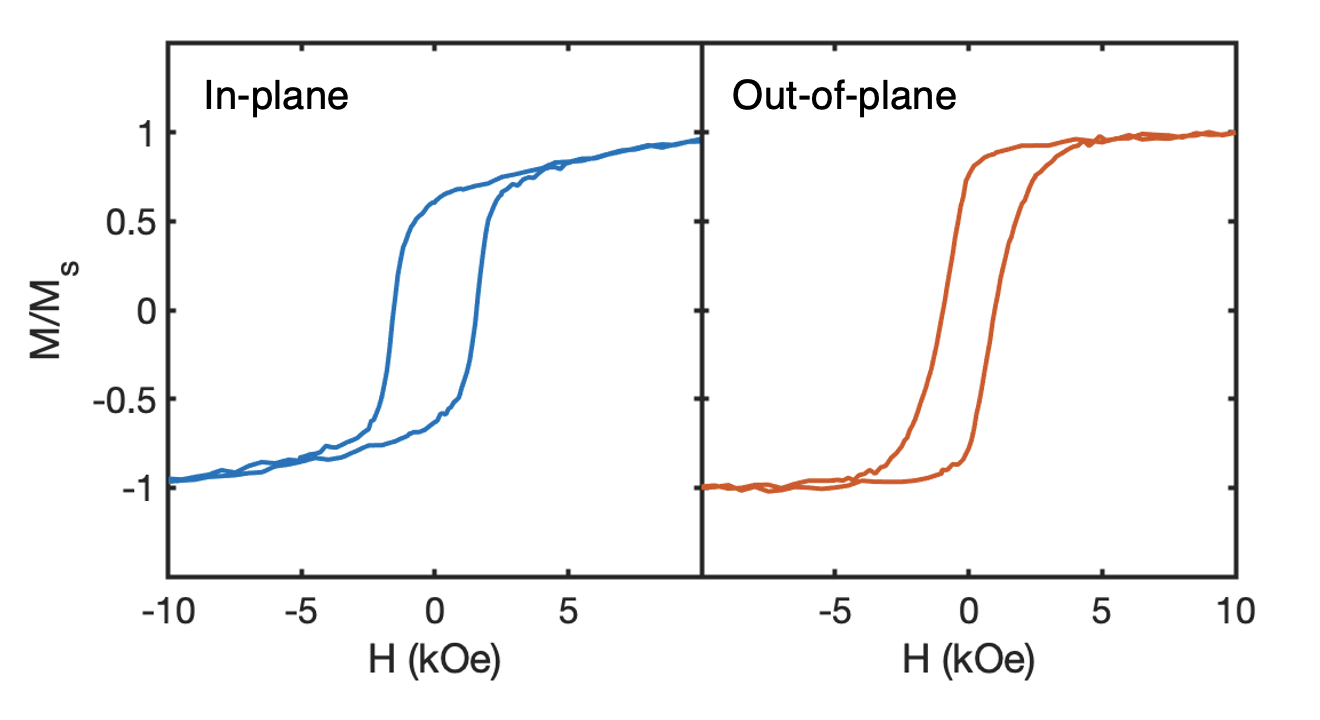


**Figure S7.** Hysteresis loops of the 20 nm (FeCoNiMnCu)Pt film sputtered using the composite target after RTA at 600 °C for 60 s for the in-plane and out-of-plane geometry.

**(FeCoNiMnCu)Pt films deposited by co-sputtering of Pt with a composite FeCoNiMnCu target**

Additional (FeCoNiMnCu)Pt films were deposited by co-sputtering of Pt with the composite target formed by powder compaction. EDX analysis of the composite target film gives an average composition of Fe_0.11_Co_0.09_Ni_0.15_Mn_0.07_Cu_0.12_Pt_0.46_, vs. Fe_0.11_Co_0.12_Ni_0.10_Mn_0.09_Cu_0.10_Pt_0.48_ for the film deposited by co-sputtering of elemental targets. Figure S6 shows the GIXRD scans ($\omega=$ 0.4$^{\circ}$) for the 20 nm (FeCoNiMnCu)Pt films deposited by composite target method after RTA treatment for 60 s at 600 $^{\circ}$C. The XRD pattern is very similar to that of Fig. 6a, indicating that the *L*1_0_ structure is readily obtained by post-annealing using either method. The composite target film has slightly smaller lattice parameters of *a* = 3.57 $Å$ and *c* = 3.75 $Å$ compared with the film sputtered using the elemental targets (*a* = 3.60 $Å$ and *c* = 3.83 $Å$), which may be due to the slight differences in composition, particularly the lower Pt content. Figure S7 shows the major hysteresis loops of the composite target sample measured using VSM for the in-plane and out-of-plane geometry, with coercivities of 1.60 kOe and 0.96 kOe, respectively. These coercivities are of comparable magnitude but somewhat smaller than the sample sputtered using only elemental targets. This decrease may be due to the difference in composition, specifically the higher Ni content, as addition of Ni in FePt is known to steadily reduce the anisotropy due to the increase in effective 3*d* electron density.^[4]^ Here, the effective electron density is increased from 9.4 in the elemental target film to 10 in the composite target film. In summary, the (FeCoNiMnCu)Pt films deposited by co-sputtering of Pt with either elemental targets or a composite target both exhibit similar structure and magnetic properties after RTA, with small differences attributed to changes in film composition. Deposition using a composite target has the advantage of requiring fewer sputtering guns. However, deposition by co-sputtering of elemental targets allows convenient and fine control over the film composition by adjusting sputtering power on each source.

**References**

[1] A. Debski, R. Debski, W. Gasior, *Arch. Metall. Mater.* **2014**, 59, 1337.

[2] N. P. Suponev, R. M. Grechishkin, M. B. Lyakhova, Y. E. Pushkar, *J. Magn. Magn. Mater.* **1996**, 157/158, 376.

[3] S. N. Hsiao, S. C. Wu, S. H. Liu, J. L. Tsai, S. K. Chen, Y. C. Chang, H. Y. Lee, *J. Magn. Magn. Mater.* **2015**, 394, 121.

[4] a) A. Sakuma, *J. Phys. Soc. Jpn.* **1994**, 63, 3053; b) J. U. Thiele, K. R. Coffey, M. F. Toney, J. A. Hedstrom, A. J. Kellock, *J. Appl. Phys.* **2002**, 91, 6595.
